# 

**Published:** 1864-03

**Authors:** 


### BOOK NOTICES AND COMMUNICATIONS DEFERRED.

From want of space in this number, we are compelled to defer till our next issue notice of the following works:

*Transactions of the Illinois State Medical Society.*

*Ellis' Medical Formulary.* New Edition.

*Nervous and Vascular Connection between the Mother and Fœtus in Utero.* By John O'Reilly, New York.

*Dalton's Human Physiology.* 3d Edition. Blanchard & Lea.

Several original communications are also reserved for our next issue.

